# Supplementary material for: A human fetal liver-derived infant MLL-AF4 acute lymphoblastic leukemia model reveals a distinct fetal gene expression program
Source: Nat Commun. 2021 Nov 25;12:6905. doi: 10.1038/s41467-021-27270-z (PMC8616957; doi:10.1038/s41467-021-27270-z)
Supplement: Supplementary file 1 — Supplementary Information [file 41467_2021_27270_MOESM1_ESM.pdf]

A human fetal liver-derived infant *MLL-AF4* Acute Lymphoblastic Leukemia model reveals a distinct fetal gene expression program

Supplementary Information

Siobhan Rice[1], Thomas Jackson[2], Nicholas T Crump[1], Nicholas Fordham[1], Natalina Elliott[2], Sorcha O'Byrne[2], Maria del Mar Lara Fanego[3], Dilys Addy[3], Trisevgeni Crabb[3], Carryl Dryden[3], Sarah Inglott[3], Dariusz Ladon[3], Gary Wright[3], Jack Bartram[3], Philip Ancliff[3], Adam J Mead[1], Christina Halsey[4,5], Irene Roberts[1,2], Thomas A Milne[*1], Anindita Roy[*1,2]

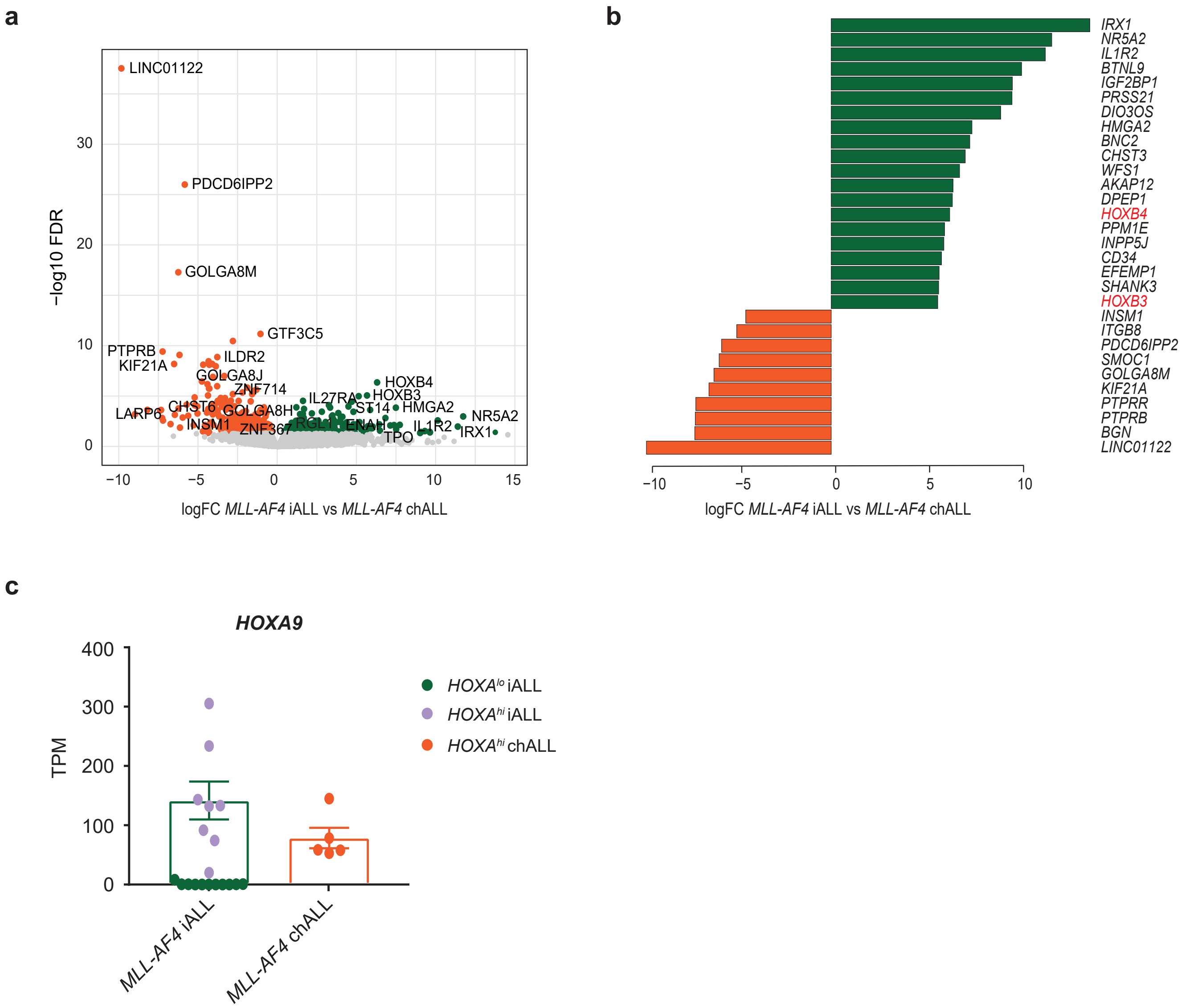
a
LINC01122
PDCD6IPP2
GOLGA8M
GTF3C5
PTPRB
KIF21A
ILDR2
GOLGA8J
ZNF714
LARP6
CHST6
GOLGA8H
INSM1
ZNF367
IL27RA
HOXB4
HOXB3
ST14
HMGA2
NR5A2
IL1R2
IRX1
TPO
−log10 FDR
logFC MLL-AF4 iALL vs MLL-AF4 chALL
b
IRX1
NR5A2
IL1R2
BTNL9
IGF2BP1
PRSS21
DIO3OS
HMGA2
BNC2
CHST3
WFS1
AKAP12
DPEP1
HOXB4
PPM1E
INPP5J
CD34
EFEMP1
SHANK3
HOXB3
INSM1
ITGB8
PDCD6IPP2
SMOC1
GOLGA8M
KIF21A
PTPRR
PTPRB
BGN
LINC01122
logFC MLL-AF4 iALL vs MLL-AF4 chALL
c
HOXA9
TPM
HOXA^lo iALL
HOXA^hi iALL
HOXA^hi chALL
MLL-AF4 iALL
MLL-AF4 chALL

Supplementary Figure 1

a. Volcano plot showing all differentially expressed genes between *MLL-AF4* infant-ALL and *MLL-AF4* childhood ALL (dark green = significantly upregulated in *MLL-AF4* infant-ALL (FDR<0.05, edgeR exact test); orange = significantly upregulated in *MLL-AF4* childhood-ALL (FDR<0.05, edgeR exact test), gray = not significantly differentially expressed (FDR>0.05, edgeR exact test)). A selection of the most differentially expressed genes are labelled.

b. Barplot showing the genes with the greatest logFC in *MLL-AF4* infant-ALL (green; top 20) and *MLL-AF4* childhood-ALL (orange; top 10).

c. Barplot showing expression of *HOXA9* in *MLL-AF4* infant-ALL (iALL, n=19) and *MLL-AF4* childhood-ALL (chALL, n=5) from a previously published patient dataset[1]. Values are shown as transcripts per million (TPM). Data shown as mean ± SEM. Patients were considered to have a *HOXA*^{lo} molecular profile when they showed a *HOXA9* expression < 15 TPM. (dark green = *HOXA*^{lo} *MLL-AF4* infant-ALL (iALL), n=11; purple = *HOXA*^{hi} *MLL-AF4* infant-ALL (iALL), n=8; orange = *HOXA*^{hi} *MLL-AF4* childhood-ALL (chALL)) (ns; p>0.5)

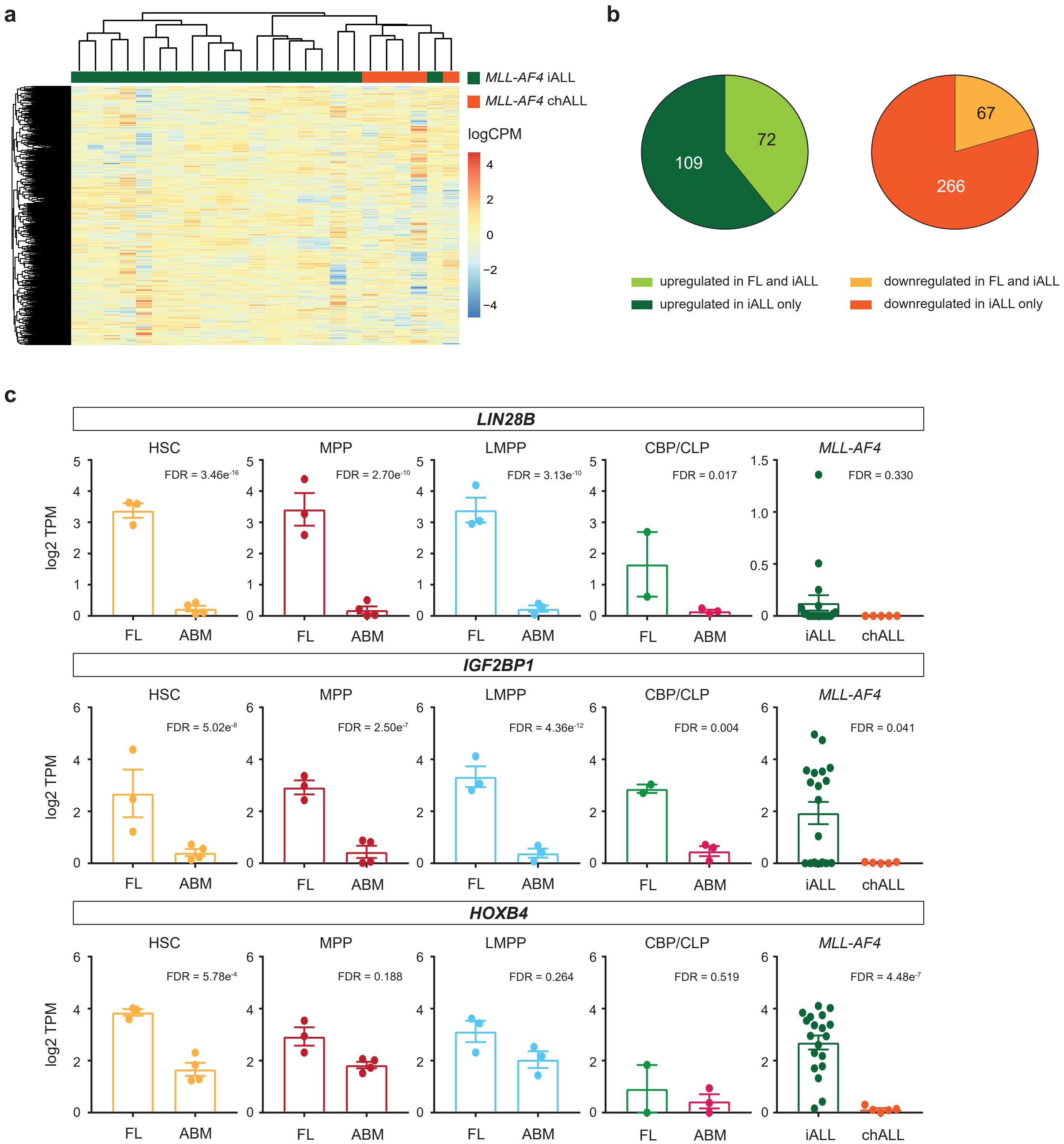
a
MLL-AF4 iALL
MLL-AF4 chALL
logCPM
4
2
0
−2
−4
b
72
109
67
266
upregulated in FL and iALL
upregulated in iALL only
downregulated in FL and iALL
downregulated in iALL only
c
LIN28B
HSC
FDR = 3.46e-16
MPP
FDR = 2.70e-10
LMPP
FDR = 3.13e-10
CBP/CLP
FDR = 0.017
MLL-AF4
FDR = 0.330
log2 TPM
FL
ABM
iALL
chALL
IGF2BP1
FDR = 5.02e-8
FDR = 2.50e-7
FDR = 4.36e-12
FDR = 0.004
FDR = 0.041
HOXB4
FDR = 5.78e-4
FDR = 0.188
FDR = 0.264
FDR = 0.519
FDR = 4.48e-7

Supplementary Figure 2

- **a.** Heatmap showing clustering of *MLL-AF4* infant-ALL (iALL (green), n=19) and *MLL-AF4* childhood-ALL (chALL (orange), n=5) based on 5,709 significantly differentially expressed genes between FL and ABM HSPCs (FDR<0.05, edgeR exact test, Supplementary Table 2). Color scale = log2 counts per million (logCPM).
- **b.** Pie charts showing proportion of genes upregulated in *MLL-AF4* infant-ALL (compared to *MLL-AF4* childhood ALL; dark green) that are also upregulated in FL (compared to ABM; light green), and the proportion of genes downregulated in *MLL-AF4* infant-ALL (compared to *MLL-AF4* childhood ALL; orange) that are also downregulated in FL (compared to ABM; yellow) (see Supplementary Table 2). Values shown as number of genes.
- **c.** Barplot showing expression of *LIN28B, IGF2BP1* and *HOXB4* in FL and ABM HSPC subpopulations (HSC = hematopoietic stem cell (n=3 FL, n=4 ABM), MPP = multipotent progenitor cell (n=3 FL, n=4 ABM), LMPP = lymphoid-primed multipotent progenitor cell (n=3 FL, n=4 ABM), CBP = committed B progenitor (n=2 FL), CLP = common lymphoid progenitor (n=4 ABM)), as well as *MLL-AF4* infant-ALL (iALL; n=19) and *MLL-AF4* childhood ALL (chALL; n=5). Data shown as mean ± SEM.

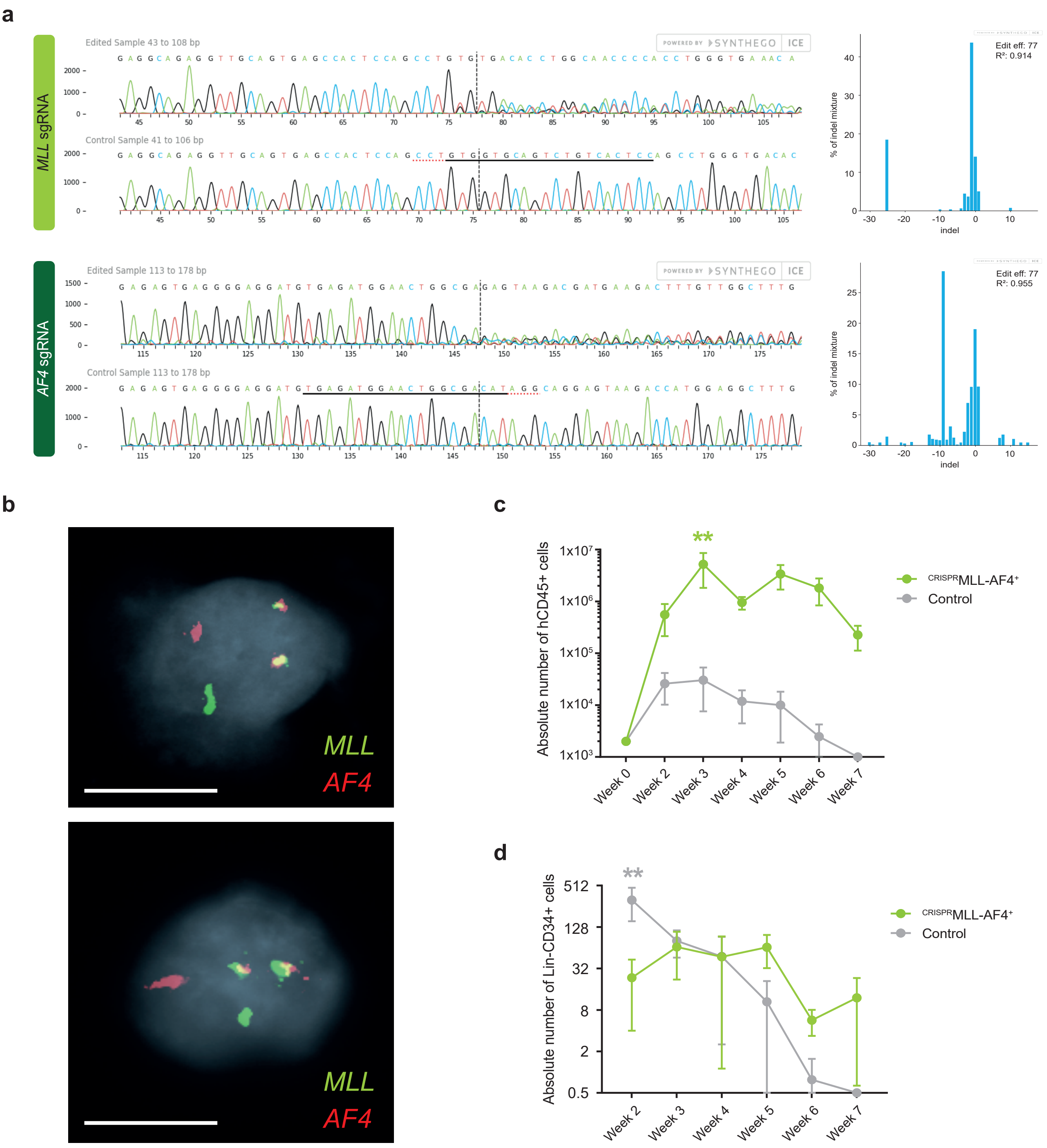
a
MLL sgRNA
Edited Sample 43 to 108 bp
Control Sample 41 to 106 bp
POWERED BY SYNTHEGO ICE
Edit eff: 77
R²: 0.914
% of indel mixture
indel
AF4 sgRNA
Edited Sample 113 to 178 bp
Control Sample 113 to 178 bp
Edit eff: 77
R²: 0.955
b
MLL
AF4
c
Absolute number of hCD45+ cells
1x10^7
1x10^6
1x10^5
1x10^4
1x10^3
Week 0
Week 2
Week 3
Week 4
Week 5
Week 6
Week 7
CRISPRMLL-AF4+
Control
d
Absolute number of Lin-CD34+ cells
512
128
32
8
2
0.5
Week 2
Week 3
Week 4
Week 5
Week 6
Week 7
CRISPRMLL-AF4+
Control

Supplementary Figure 3

a. Synthego ICE Analysis (https://ice.synthego.com/) results for individual sgRNA efficiency tests for *MLL*-sgRNA and *AF4*-sgRNA in FL CD34+ cells. (left) Sanger sequencing tracks for edited cells (top) and unedited controls (bottom) around the PAM site. (right) Quantification of indels in edited cells. *MLL*-sgRNA and *AF4*-sgRNA both showed an editing efficiency of 77%.

b. Representative *MLL-AF4* FISH images for 2 biological replicates of ^{CRISPR}*MLL-AF4*+ cells *in vitro* (donors 4 (top) and 5 (bottom)). Cells taken at week 4. Scale bar = 10μm.

c. Cumulative absolute number of human CD45+ cells per well over time during MS-5 co-culture assay of ^{CRISPR}*MLL-AF4*+ and control cells (n=3; donor 1-3). ** p=0.0015 (Two-way ANOVA with Sidak correction for multiple comparisons). Data shown as mean ± SEM.

d. Cumulative absolute number of human Lin-CD34+ cells per well over time during MS-5 co-culture assay of ^{CRISPR}*MLL-AF4*+ and control cells (n=3; donor 1-3). ** p=0.0046 (Two-way ANOVA with Sidak correction for multiple comparisons). Data shown as mean ± SEM.

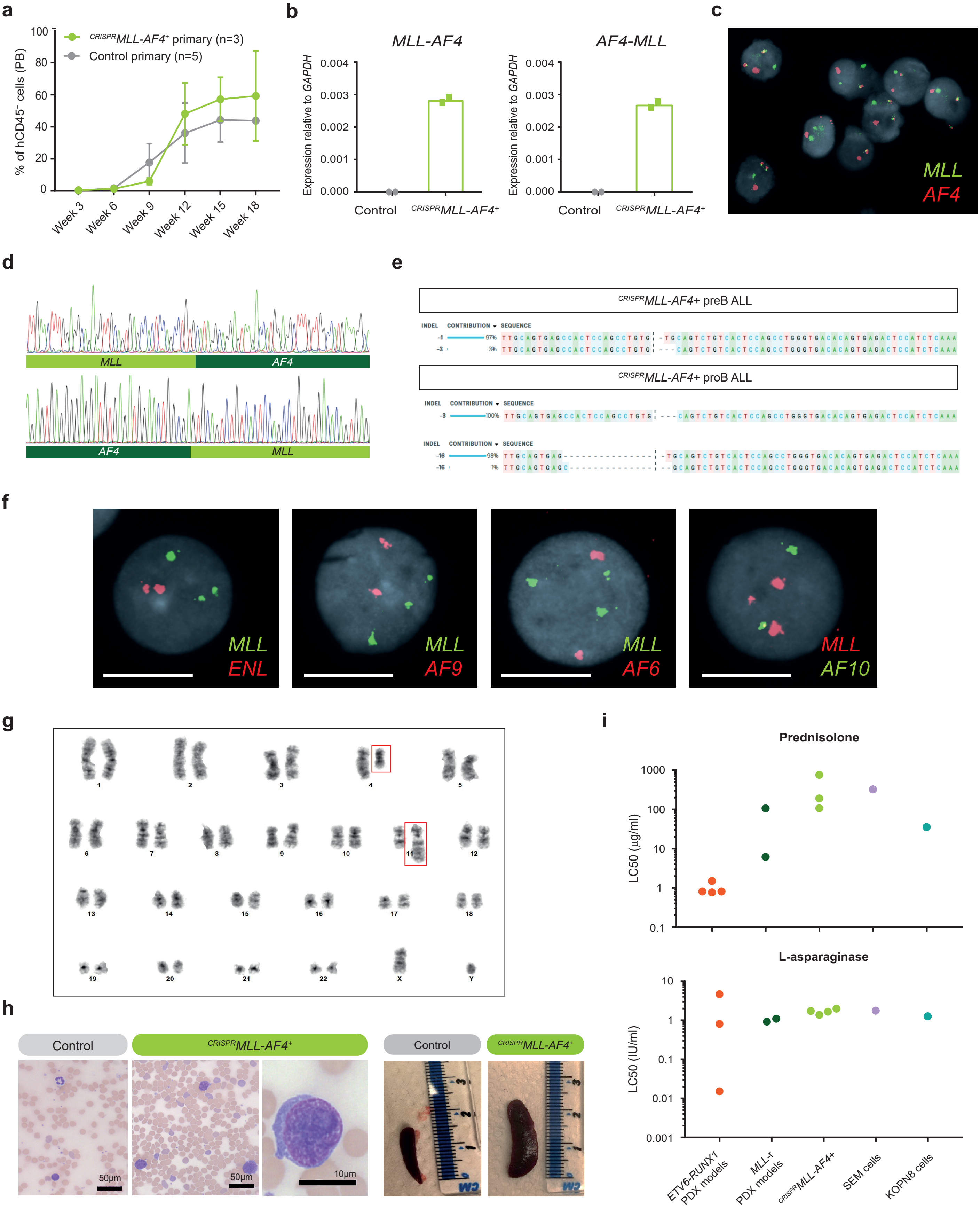

a
CRISPRMLL-AF4+ primary (n=3)
Control primary (n=5)
% of hCD45+ cells (PB)
100
80
60
40
20
0
Week 3
Week 6
Week 9
Week 12
Week 15
Week 18
b
MLL-AF4
AF4-MLL
Expression relative to GAPDH
0.004
0.003
0.002
0.001
0.000
Control
CRISPRMLL-AF4+
c
MLL
AF4
d
MLL
AF4
AF4
MLL
e
CRISPRMLL-AF4+ preB ALL
INDEL
CONTRIBUTION
SEQUENCE
CRISPRMLL-AF4+ proB ALL
f
MLL
ENL
MLL
AF9
MLL
AF6
MLL
AF10
g
1
2
3
4
5
6
7
8
9
10
11
12
13
14
15
16
17
18
19
20
21
22
X
Y
h
Control
CRISPRMLL-AF4+
50µm
50µm
10µm
Control
CRISPRMLL-AF4+
i
Prednisolone
LC50 (µg/ml)
1000
100
10
1
0.1
L-asparaginase
LC50 (IU/ml)
10
1
0.1
0.01
0.001
ETV6-RUNX1 PDX models
MLL-r PDX models
CRISPRMLL-AF4+
SEM cells
KOPN8 cells

Supplementary Figure 4

- **a.** PB engraftment of human CD45+ (hCD45+) cells over time in primary ^{CRISPR}*MLL-AF4*+ (n=3) and control (n=5) recipient mice. Quantified as a percentage of all CD45+ cells (mouse CD45.1+ and human CD45+). Data shown as mean ± SEM (Two-way ANOVA with Sidak correction for multiple comparisons. ns; p>0.5).
- **b.** RT-qPCR showing expression of *MLL-AF4* (n=2) and *AF4-MLL* (n=2) relative to *GAPDH* in human CD45+ cells isolated from PB at week 12 post-engraftment.
- **c.** Representative *MLL-AF4* FISH image showing multiple nuclei for ^{CRISPR}*MLL-AF4*+ ALL spleen (n=3; donors 1-2).
- **d.** Sanger sequencing tracks showing *MLL-AF4* and *AF4-MLL* genomic DNA breakpoints in blasts isolated from the spleen of ^{CRISPR}*MLL-AF4*+ mice. Breakpoint regions were amplified by PCR before Sanger sequencing in order to examine the translocated allele without contamination by the remaining WT allele. *MLL* and *AF4* portions are labelled below each track.
- **e.** Sanger sequencing data showing indels present in each ^{CRISPR}*MLL-AF4*+ ALL (n=3) compared to original unedited FL cells (donors 1 and 2). Contribution = percentage of cells with each indel. Deletions are shown as horizontal dashed lines in the sequence. The CRISPR-Cas9 cut sites is shown as a vertical dashed line in the sequence.
- **f.** Representative FISH for the top 4 other *MLL* translocations (*MLL-ENL, MLL-AF9, MLL-AF6* and *MLL-AF10*) in the ^{CRISPR}*MLL-AF4*+ ALL spleen (n=3; donors 1-2). 3 signals can be detected for *MLL* in each case due to the presence of the *MLL-AF4*/t(4;11) translocation. Scale bar = 10μm.
- **g.** Representative karyotype data for ^{CRISPR}*MLL-AF4*+ ALL (n=1; preB ALL).
- **h.** (left) Representative H&E-stained peripheral blood (PB) films for control (left) and ^{CRISPR}*MLL-AF4*+ (right) primary recipient mice (n=5; donors 1, 2, 3 and 6). Low magnification images (scale bar = 50μm) show multi-lineage cells in the PB in controls

and predominantly circulating blast cells in ^{CRISPR}*MLL-AF4*+ mice. High magnification image (scale bar = 10μm) shows a representative blast cell from ^{CRISPR}*MLL-AF4*+ PB. (right) Representative images of the spleens of control and ^{CRISPR}*MLL-AF4*+ mice.

i. Resistance (represented by LC50) to prednisolone (μg/ml) and L-asparaginase (IU/ml) for *ETV6-RUNX1* patient-derived xenograft (PDX) models, *MLL*-r PDX models, ^{CRISPR}*MLL-AF4*+ ALL (preB secondary recipients and proB primary and secondary recipients) and the SEM and KOPN8 cell lines. Each point represents a biological replicate.

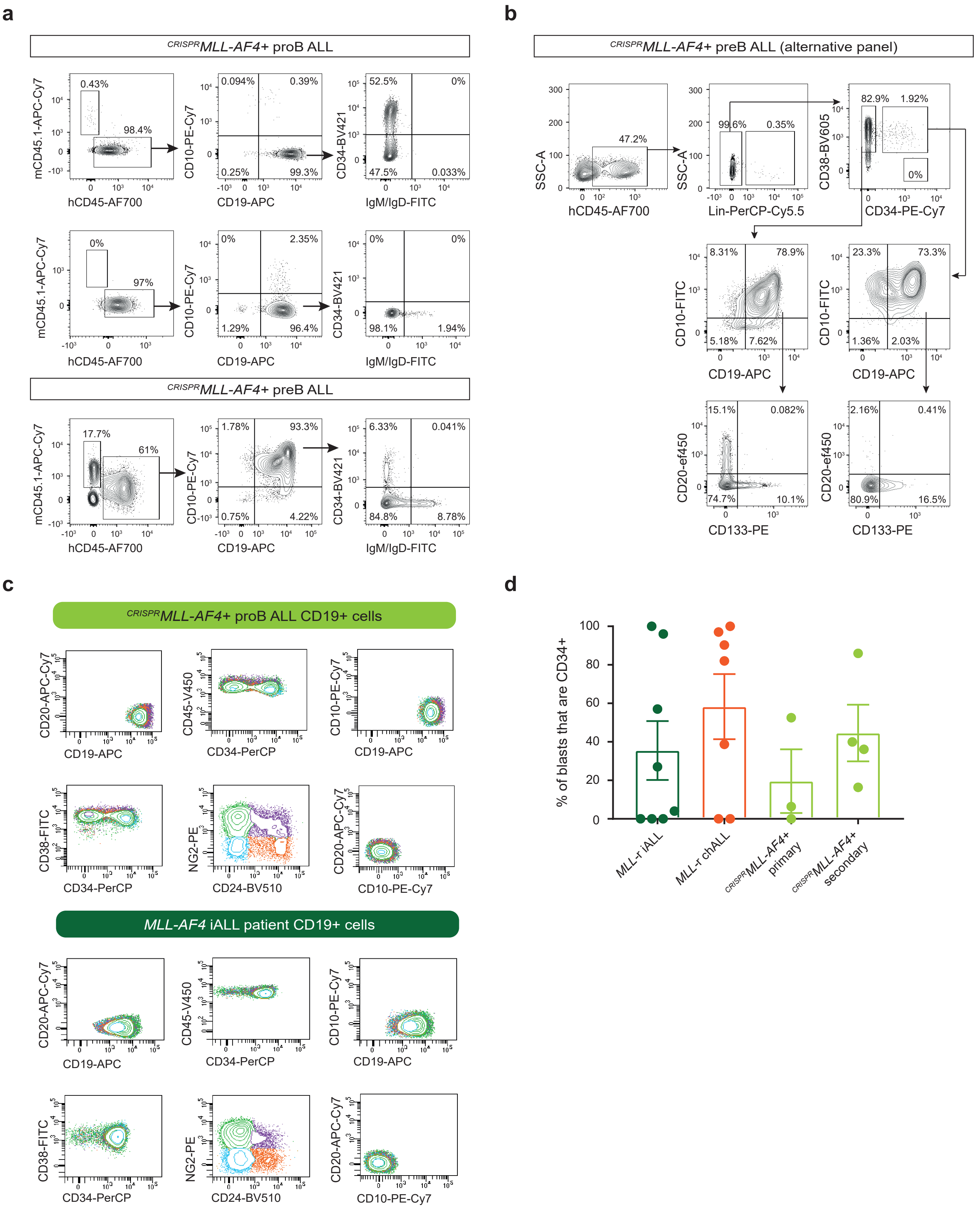
a
CRISPRMLL-AF4+ proB ALL
mCD45.1-APC-Cy7
hCD45-AF700
0.43%
98.4%
CD10-PE-Cy7
CD19-APC
0.094%
0.39%
0.25%
99.3%
CD34-BV421
IgM/IgD-FITC
52.5%
0%
47.5%
0.033%
0%
97%
0%
2.35%
1.29%
96.4%
0%
0%
98.1%
1.94%
CRISPRMLL-AF4+ preB ALL
17.7%
61%
1.78%
93.3%
0.75%
4.22%
6.33%
0.041%
84.8%
8.78%
b
CRISPRMLL-AF4+ preB ALL (alternative panel)
SSC-A
hCD45-AF700
47.2%
Lin-PerCP-Cy5.5
99.6%
0.35%
CD38-BV605
CD34-PE-Cy7
82.9%
1.92%
0%
CD10-FITC
CD19-APC
8.31%
78.9%
5.18%
7.62%
23.3%
73.3%
1.36%
2.03%
CD20-ef450
CD133-PE
15.1%
0.082%
74.7%
10.1%
2.16%
0.41%
80.9%
16.5%
c
CRISPRMLL-AF4+ proB ALL CD19+ cells
CD20-APC-Cy7
CD19-APC
CD45-V450
CD34-PerCP
CD10-PE-Cy7
CD38-FITC
NG2-PE
CD24-BV510
CD10-PE-Cy7
MLL-AF4 iALL patient CD19+ cells
d
% of blasts that are CD34+
100
80
60
40
20
0
MLL-r iALL
MLL-r chALL
CRISPRMLL-AF4+ primary
CRISPRMLL-AF4+ secondary

Supplementary Figure 5

a. Representative flow cytometry plots of viable, single cells in proB ^{CRISPR}*MLL-AF4*+ (top) and preB ^{CRISPR}*MLL-AF4*+ (bottom) BM at termination. (mCD45.1 = mouse CD45; hCD45 = human CD45).

b. Representative extended flow cytometry plots of viable, single cells in preB ^{CRISPR}*MLL-AF4*+ BM at termination.

c. Representative flow cytometry plots of CD19+ blasts from ^{CRISPR}*MLL-AF4*+ BM at termination (week 18) (top) and an *MLL-AF4* infant-ALL patient BM (bottom). Datapoints are colored in all plots based on surface NG2 and CD24 expression.

d. Barplot showing the proportion of CD19+ B-ALL blasts that are CD34+ for primary *MLL*-r infant-ALL (dark green, n=8) and *MLL*-r childhood-ALL (orange, n=7) patient samples, and primary (n=3) and secondary (n=4) ^{CRISPR}*MLL-AF4*+ ALL (light green). Data shown as mean ± SEM (One-way ANOVA with Tukey correction for multiple comparisons. ns; p>0.5).

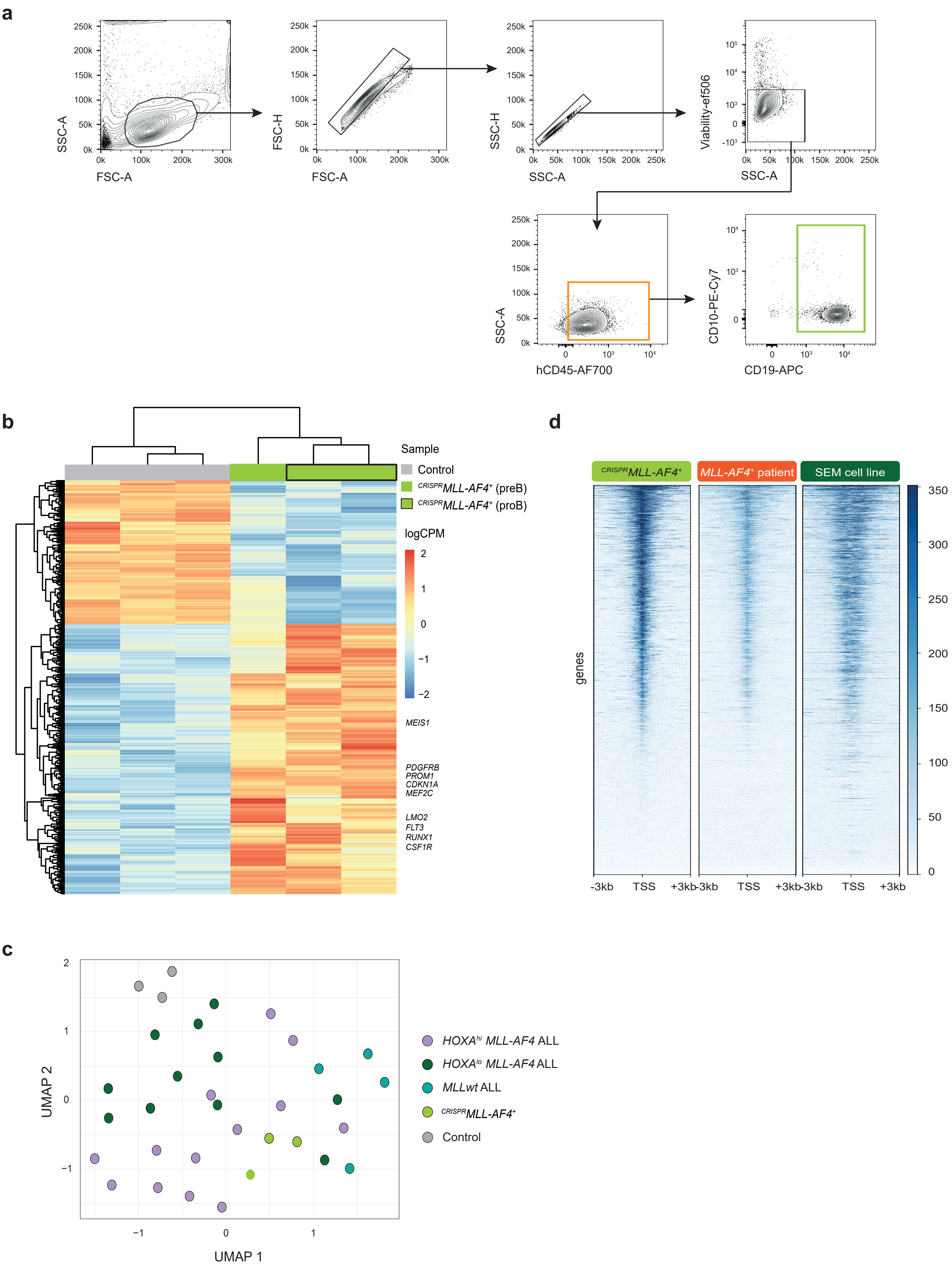
a
SSC-A
FSC-A
FSC-H
FSC-A
SSC-H
SSC-A
Viability-ef506
SSC-A
SSC-A
hCD45-AF700
CD10-PE-Cy7
CD19-APC
b
Sample
Control
CRISPR MLL-AF4+ (preB)
CRISPR MLL-AF4+ (proB)
logCPM
MEIS1
PDGFRB
PROM1
CDKN1A
MEF2C
LMO2
FLT3
RUNX1
CSF1R
c
UMAP 2
UMAP 1
HOXAhi MLL-AF4 ALL
HOXAlo MLL-AF4 ALL
MLLwt ALL
CRISPR MLL-AF4+
Control
d
CRISPR MLL-AF4+
MLL-AF4+ patient
SEM cell line
genes
-3kb
TSS
+3kb

Supplementary Figure 6

- **a.** Representative sort strategy to sort CD19+ cells from ^{CRISPR}*MLL-AF4*+ and control primary mouse BM for qPCR analysis (orange; hCD45+) and RNA-seq analysis (light green; hCD45+CD19+) (n=3 ^{CRISPR}*MLL-AF4*+ and n=3 control, donors 1-2).
- **b.** Heatmap showing significantly differentially expressed genes (1,068 genes, FDR < 0.05) between primary control (grey) and ^{CRISPR}*MLL-AF4*+ (light green; black border = proB, no border = preB) mice. A selection of genes known to be upregulated in *MLL-AF4* ALL are labelled.
- **c.** UMAP showing clustering of ^{CRISPR}*MLL-AF4*+ (light green; black border = proB, no border = preB) and control (gray) mice with *HOXA*^{lo} *MLL-AF4* ALL (iALL; dark green), *HOXA*^{hi} *MLL-AF4* ALL (iALL; purple) and *MLL*wt (blue) ALL patients from a publicly available dataset[20] based on 5,785 significantly differentially expressed genes between ^{CRISPR}*MLL-AF4*+ ALL, control, *MLL-AF4* ALL and *MLL*wt ALL (FDR<0.05, edgeR glm test).
- **d.** Heatmap showing MLL-N ChIP-seq enrichment for a 6kb region centered on the promoter (transcriptional start site (TSS)) of all genes in ^{CRISPR}*MLL-AF4*+ ALL, the SEM cell line and a primary *MLL-AF4* childhood-ALL (chALL) patient sample, sorted by MLL-N ChIP-seq signal in ^{CRISPR}*MLL-AF4*+ ALL. Scale = reads/bp/10^7 total reads.

**Supplementary Table 1: Analysis of off-target editing**					
			**% of Sanger sequencing reads matching wild-type, unedited sequence**		
**gene name**	**PAM location**	**sgRNA**	**primary** ^{CRISPR}***MLL-AF4+*** **ALL 1 (preB)**	**primary** ^{CRISPR}***MLL-AF4+*** **ALL 2 (proB)**	**primary** ^{CRISPR}***MLL-AF4+*** **ALL 3 (proB)**
SMR3A	intron	MLL	100%	100%	100%
CTNNDA	intron	MLL	100%	100%	100%
PARVA	intron	MLL	100%	100%	100%
KDM6B	intron	MLL	100%	100%	100%
DCC	intron	MLL	100%	100%	100%
TRGC2	intron	MLL	100%	100%	100%
ATRNL1	intron	MLL	100%	100%	100%
FOXO1	intron	AF4	100%	100%	100%
KCNQ2	exon	AF4	100%	100%	100%
MLL wild-type allele	intron	MLL	0% (100% with 1bp deletion)	0% (100% with 3bp deletion)	1% (99% with 16bp deletion)

**Supplementary Table 2: Summary of *in vivo* experiments**						
	**Primary**		**Secondary**		**Tertiary**	
	**Control**	^{CRISPR}***MLL-AF4+***	**Control**	^{CRISPR}***MLL-AF4+***	**Control**	^{CRISPR}***MLL-AF4+***
Engrafted	5/5	3/3	1/1	4/4	2/2	3/3
ALL	0/5	3/3	0/1	4/4	0/2	3/3
Median latency	n/a	18 weeks	n/a	11.5 weeks	n/a	8 weeks
BM engraftment	5/5	3/3	1/1	4/4	2/2	3/3
Splenomegaly	0/5	3/3	0/1	4/4	0/2	3/3
CNS infiltration (/of those tested)	0/1	1/1	0/1	3/3	ND	ND
Pred resistant *in vitro* (LC50 > 100µg/ml)	n/a	1/1	n/a	2/2	ND	ND
L-asp resistant *in vitro* (LC50 > 0.1 IU/ml)	n/a	1/1	n/a	3/3	ND	ND
proB (CD19+CD10-CD20-IgM/IgD-)	n/a	2/3	n/a	2/4	n/a	3/3
preB (CD19+CD10+CD20-IgM/IgD-)	n/a	1/3	n/a	2/4	n/a	0/3
CD34 positive (>20% of blasts)	n/a	ProB (1/2); PreB (0/1)	n/a	ProB (1/2); PreB (2/2)	n/a	ND
Karyotype	ND	donor 1: 46 XY t(4;11); donor 2: 45 XY t(4;11) der(14;21)(q10;q10)	ND	ND	ND	ND
MLL-AF4/t(4;11) positive by FISH	n/a	80-97% (n=3, donor 1 and 2)	ND	ND	ND	ND
MLL-AF4 expression	0/2	2/2	ND	ND	ND	ND
AF4-MLL expression	0/2	2/2	ND	ND	ND	ND
VDJ rearrangement	ND	2/3 (ProB and PreB) clonal; 1/3 (ProB) non-rearranged	ND	ND	ND	ND

**Supplementary Table 3: Oligonucleotides used**		
**sgRNAs**		
**Name**	**Sequence**	
MLL-sgRNA	GGAGUGACAGACUGCACCAC	
AF4-sgRNA	UGAGAUGGAACUGGCGACAU	
**Primers**		
**Name**	**Forward sequence**	**Reverse sequence**
^{CRISPR}*MLL-AF4+ MLL-AF4* breakpoint (gDNA)	AGGTGTGGTGGTGGGC	GCCCTATCAGTTCCCTGCCA
^{CRISPR}*MLL-AF4+ AF4-MLL* breakpoint (gDNA)	GAGAGGCTGAGGTTGTAGGG	GGCAACTGATTACACCCAATT
^{CRISPR}*MLL-AF4+ MLL-AF4* (cDNA)	GTGGAAGGCAACATCAGGC	TATTGCTGTCAAAGGAGGCGG
^{CRISPR}*MLL-AF4+ AF4-MLL* (cDNA)	CAGAAGCCCACGGCTTATGT	AGGCACTCAGGGTGATAGC

**Supplementary Table 4: Antibodies used**			
**Antibodies**	**Dilution**	**Company**	**Catalog #**
**Flow Cytometry**			
Viability eflour506	1:100	eBioscience	65-0866-18
mouse CD45.1 APC-Cy7	1:50	Biolegend	103116
CD45 AF700	1:100	eBioscience	56-9459-42
CD2 PerCP-Cy5.5	1:50	Biolegend	300216
CD3 PerCP-Cy5.5	1:50	Biolegend	317336
CD3 BV711	1:200	Biolegend	317327
CD14 PerCP-Cy5.5	1:50	Biolegend	301824
CD16 PerCP-Cy5.5	1:50	Biolegend	302028
CD56 PerCP-Cy5.5	1:50	Biolegend	318322
CD56 BV605	1:50	Biolegend	318334
CD235a PerCP-Cy5.5	1:100	Biolegend	318322
CD34 BV421	1:50	Biolegend	343610
CD34 PE-Cy7	1:50	eBioscience	25-0349-42
CD38 BV605	1:100	Biolegend	303532
CD19 APC	1:100	Biolegend	302212
CD10 FITC	1:20	eBioscience	11-0106-42
CD10 PE-Cy7	1:50	eBioscience	25-0106-42
CD20 eflou450	1:50	eBioscience	48-0209-42
IgM FITC	1:50	Biolegend	314506
IgD FITC	1:50	biolegend	348205
CD133 PE	1:100	Miltenyi	130-080-801
CD24 BV510	2.5-5:100 (batch titration-dependent)	Pharmagen	563035
CD45 V450	2.5-5:100 (batch titration-dependent)	BD	642275
CD34 PerCp	2.5-5:100 (batch titration-dependent)	BD	340909
CD38 FITC	2.5-5:100 (batch titration-dependent)	BD	340909
NG2 PE	2.5-5:100 (batch titration-dependent)	Beckman Coulter	B92429
**ChIP**			
MLL-N	1:500	Bethyl	A300-086A
AF4-C	1:500	Abcam	ab31812
H3K79me2	1:500	Millipore	04-835

Supplementary Table 5: Commercial products used		
**Product**	**Company**	**Catalog #**
MLL-AF4 FISH probes	Cytocell	LPH 081
MLL-ENL FISH probes	Cytocell	RU-LPH 082
MLL-AF9 FISH probes	Cytocell	RU-LPH 083
MLL-AF6 FISH probes	Cytocell	RU-LPH 084
MLL-AF10 FISH probes	Cytocell	MPH 4850
RNeasy mini kit	QIAGEN	74104
DNeasy Blood and Tissue kit	QIAGEN	69506
SuperScript III reverse transcriptase kit	Invitrogen	18080051
AMPure XP	Beckman Coulter	A63880
NEBNext Ultra^{TM} II DNA Library Prep Kit	New England Biolabs	E7645
NEBNext Ultra^{TM} Directional RNA Library Prep Kit	New England Biolabs	E7420
Cell Proliferation Kit I	Roche	11465007001
Alt-R® S.p. Cas9 Nuclease V3, 100 µg	Integrated DNA Technologies	1081058
Neon™ Transfection System	Invitrogen	MPK5000
Neon™ Transfection System Pipette	Invitrogen	MPP100
Neon™ Transfection System 10 µL Kit	Invitrogen	MPK1025
StemLine II	Sigma	S0192
a-MEM	Gibco	41061-029
Fetal Bovine Serum (FBS)	Sigma	F7524
SCF	Peprotech	HHSC3
FLT3L	Peprotech	HHSC3
IL2	Peprotech	200-02
IL7	Peprotech	200-07
TPO	Peprotech	HHSC3
HEPES	Gibco	15630-056
2-Mercaptoethanol	Sigma	M3148
Penicillin/Streptomycin	Sigma	P4333-100ML
L-glutamine	Gibco	25030-024
Trypsin	Gibco	10779413
EDTA	Gibco	15575020
