# Supplementary material for: A human fetal liver-derived infant MLL-AF4 acute lymphoblastic leukemia model reveals a distinct fetal gene expression program
Source: Nat Commun. 2021 Nov 25;12:6905. doi: 10.1038/s41467-021-27270-z (PMC8616957; doi:10.1038/s41467-021-27270-z)
Supplement: Supplementary file 3 — Description of Additional Supplementary Files [file 41467_2021_27270_MOESM3_ESM.pdf]

A human fetal liver-derived infant *MLL-AF4* Acute Lymphoblastic Leukemia model reveals a distinct fetal gene expression program

Description of Additional Supplementary Files

Supplementary Data 1: Differentially expressed genes in *MLL-AF4* infant-ALL vs *MLL-AF4* childhood-ALL. List of genes differentially expressed in *MLL-AF4* infant-ALL (n=11) compared to *MLL-AF4* childhood-ALL (n=5)[1] (edgeR exact test; FDR<0.05), including log fold change (logFC) log counts per million (logCPM), P value and adjusted P value (FDR) for all genes.

Supplementary Data 2: Combined table of differentially expressed genes in FL HSPC vs ABM HSPC, and in *MLL-AF4* infant-ALL vs *MLL-AF4* childhood-ALL. List of genes differentially expressed in FL HSPC populations[2] compared to equivalent ABM HSPC populations[3] (HSC, MPP, LMPP and CBP/CLP), and/or in *MLL-AF4* infant-ALL compared to *MLL-AF4* childhood ALL[1]. Each FL HSPC subpopulation and MLL-AF4 infant-ALL has its own column describing the effect in that subpopulation. “up” = FDR<0.05 and logFC>0, “down” = FDR<0.05 and logFC<0, “unchanged” = FDR>0.05. Data can be filtered by whether a gene is differentially expressed in 0-4 FL HSPC subpopulations analyzed.

Supplementary Data 3: Significantly differentially expressed genes between *HOXA*^{hi} *MLL-AF4* childhood-ALL, *HOXA*^{hi} *MLL-AF4* infant-ALL and *HOXA*^{lo} *MLL-AF4* infant-ALL. List of genes found to be differentially expressed following a 3-way comparison between *HOXA*^{hi} *MLL-AF4* childhood-ALL, *HOXA*^{hi} *MLL-AF4* infant-ALL and *HOXA*^{lo} *MLL-AF4* infant-ALL[1] (edgeR glm test, FDR<0.05). logFC values are given for each subtype.

Supplementary Data 4: MLL-AF4-bound genes. List of genes that have at least one MLL-AF4 binding site (defined as overlapping MLL-N and AF4-C ChIP-seq peaks) anywhere in the gene body in ^{CRISPR}*MLL-AF4*+ ALL, the SEM cell line[4] and/or an *MLL-AF4* childhood-ALL patient[5]. Each sample has its own column describing whether an MLL-AF4 binding site is present. "yes" = an MLL-AF4 binding site is present in this gene in this particular sample. "no" = an MLL-AF4 binding site is not present in this gene in this particular sample.

References

1 Andersson, A. K. *et al.* The landscape of somatic mutations in infant MLL-rearranged acute lymphoblastic leukemias. *Nat Genet* **47**, 330-337, doi:10.1038/ng.3230 (2015).

2 Agraz-Doblas, A. *et al.* Unravelling the cellular origin and clinical prognostic markers of infant B-cell acute lymphoblastic leukemia using genome-wide analysis. *Haematologica*, doi:10.3324/haematol.2018.206375 (2019).

3 Corces, M. R. *et al.* Lineage-specific and single-cell chromatin accessibility charts human hematopoiesis and leukemia evolution. *Nat Genet* **48**, 1193-1203, doi:10.1038/ng.3646 (2016).

4 Kerry, J. *et al.* MLL-AF4 Spreading Identifies Binding Sites that Are Distinct from Super-Enhancers and that Govern Sensitivity to DOT1L Inhibition in Leukemia. *Cell Rep* **18**, 482-495, doi:10.1016/j.celrep.2016.12.054 (2017).

5 Harman, J. R. *et al.* A KMT2A-AFF1 gene regulatory network highlights the role of core transcription factors and reveals the regulatory logic of key downstream target genes. *Genome Res*, doi:10.1101/gr.268490.120 (2021).
