# Supplementary material for: A human fetal liver-derived infant MLL-AF4 acute lymphoblastic leukemia model reveals a distinct fetal gene expression program
Source: Nat Commun. 2021 Nov 25;12:6905. doi: 10.1038/s41467-021-27270-z (PMC8616957; doi:10.1038/s41467-021-27270-z)
Supplement: Supplementary file 8 — Reporting Summary [file 41467_2021_27270_MOESM8_ESM.pdf]

nature research

Corresponding author(s): Anindita Roy and Thomas Milne

Last updated by author(s): Oct 29, 2021

Reporting Summary

Nature Research wishes to improve the reproducibility of the work that we publish. This form provides structure for consistency and transparency in reporting. For further information on Nature Research policies, see our Editorial Policies and the Editorial Policy Checklist.

Statistics

For all statistical analyses, confirm that the following items are present in the figure legend, table legend, main text, or Methods section.

n/a	Confirmed	
☐	☒	The exact sample size (*n*) for each experimental group/condition, given as a discrete number and unit of measurement
☐	☒	A statement on whether measurements were taken from distinct samples or whether the same sample was measured repeatedly
☐	☒	The statistical test(s) used AND whether they are one- or two-sided<br>*Only common tests should be described solely by name; describe more complex techniques in the Methods section.*
☐	☒	A description of all covariates tested
☐	☒	A description of any assumptions or corrections, such as tests of normality and adjustment for multiple comparisons
☐	☒	A full description of the statistical parameters including central tendency (e.g. means) or other basic estimates (e.g. regression coefficient) AND variation (e.g. standard deviation) or associated estimates of uncertainty (e.g. confidence intervals)
☐	☒	For null hypothesis testing, the test statistic (e.g. *F*, *t*, *r*) with confidence intervals, effect sizes, degrees of freedom and *P* value noted<br>*Give P values as exact values whenever suitable.*
☒	☐	For Bayesian analysis, information on the choice of priors and Markov chain Monte Carlo settings
☒	☐	For hierarchical and complex designs, identification of the appropriate level for tests and full reporting of outcomes
☒	☐	Estimates of effect sizes (e.g. Cohen's *d*, Pearson's *r*), indicating how they were calculated

Our web collection on statistics for biologists contains articles on many of the points above.

Software and code

Policy information about availability of computer code

Data collection	Flow cytometry data was collected using BD FACSDiva (version 8.0.2). qPCR data was collected using Quantstudio (Thermofisher Scientific). High throughput sequencing data was collected via Basespace (Illumina).
Data analysis	Flow cytometry was analyzed using FlowJo software (version 10.7.1). Sanger sequencing data were analyzed for indels using the Synthego ICE analysis online tool (https://ice.synthego.com/#/).<br>For RNA-seq analysis, following QC analysis with fastQC (http://www.bioinformatics.babraham.ac.uk/projects/fastqc; version 0.11.5) reads were aligned against the human genome assembly (hg38) using STAR (version 2.7.3a). Gene expression levels were quantified as read counts using the featureCounts function from the Subread package (version 2.0.0) with default parameters. The read counts were used to identify differential gene expression between conditions using the edgeR package (version 3.13) and to generate TPM values.<br>For ChIP-seq, quality control of FASTQ reads, genome alignment, PCR duplicate filtering, blacklisted region filtering and UCSC data hub generation was performed using an in-house pipeline which is available via Telenius et al., 2018 (Reference 53 in manuscript). Directories of sequence tags (reads) were generated from the bam files using the Homer package (version 4.10) tool makeTagDirectory. The makeBigWig.pl command was used to generate bigwig files for visualisation in UCSC, normalising tag counts to tags per 10 million. Peaks were called using the Homer tool findPeaks, with the input track provided for background correction, using the -style histone or -style factor options to call peaks in histone modification or MLL-N/AF4-C datasets, respectively.<br>All plots were generated wither either GraphPad Prism (version 7.04) or R (version 4.0.1).

For manuscripts utilizing custom algorithms or software that are central to the research but not yet described in published literature, software must be made available to editors and reviewers. We strongly encourage code deposition in a community repository (e.g. GitHub). See the Nature Research guidelines for submitting code & software for further information.

Data

Policy information about availability of data

All manuscripts must include a data availability statement. This statement should provide the following information, where applicable:
- Accession codes, unique identifiers, or web links for publicly available datasets
- A list of figures that have associated raw data
- A description of any restrictions on data availability

All newly-generated high throughput data have been deposited in the Gene Expression Omnibus (GEO) under the accession number GSE162041, which will be made available upon publication.
Listed below are the datasets associated with each figure:
Figure 1: FL bulk RNA-seq (HSC, MPP, LMPP, CBP) in triplicate
Figure 4: CRISPRMLL-AF4+ ALL and control bulk RNA-seq in triplicate, CRISPRMLL-AF4+ ALL ChIP-seq (MLL-N, AF4-C)
Figure 5: H3K79me2 ChIP-seq in CRISPRMLL-AF4+ ALL and biologically matched, unedited FL CD19+ cells
Extended Data Figure 2: FL bulk RNA-seq (HSC, MPP, LMPP, CBP) in triplicate
Extended Data Figure 5: CRISPRMLL-AF4+ ALL and control bulk RNA-seq in triplicate

Field-specific reporting

Please select the one below that is the best fit for your research. If you are not sure, read the appropriate sections before making your selection.

[x] Life sciences [ ] Behavioural & social sciences [ ] Ecological, evolutionary & environmental sciences

For a reference copy of the document with all sections, see nature.com/documents/nr-reporting-summary-flat.pdf

Life sciences study design

All studies must disclose on these points even when the disclosure is negative.

Sample size	Statistical methods were not used to assign sample size. In vitro and in vivo experiments were carried out in triplicate.
Data exclusions	No data were excluded from this analysis.
Replication	All in vitro data (cell growth assays, immunophenotyping and fusion gene qPCR) were carried out in triplicate unless otherwise stated in the manuscript. All attempts at replication were successful.<br>Primary NSG transplant data represents n=8 (CRISPRMLL-AF4+, n=3; control, n=5) animal replicates, derived from n=4 edited primary human FL samples.<br>ChIP-seq data represent a single biological replicate.<br>RNA-seq experiments were conducted in triplicate, with averaged data presented. Statistical difference between treatments were assessed using EdgeR.
Randomization	Randomization was not used in this study. All recipient mice used were 8-12 week old female NSG mice to control for recipient mouse covariates. Where possible, littermates were used as controls.
Blinding	Investigators were not blinded to experiments, however Biomedical Services staff monitoring the xenografted mice for development of disease, were blinded to the control and leukemic groups.

Reporting for specific materials, systems and methods

We require information from authors about some types of materials, experimental systems and methods used in many studies. Here, indicate whether each material, system or method listed is relevant to your study. If you are not sure if a list item applies to your research, read the appropriate section before selecting a response.

Materials & experimental systems

n/a	Involved in the study	
[ ]	[x]	Antibodies
[x]	[ ]	Eukaryotic cell lines
[x]	[ ]	Palaeontology and archaeology
[ ]	[x]	Animals and other organisms
[ ]	[x]	Human research participants
[x]	[ ]	Clinical data
[x]	[ ]	Dual use research of concern

Methods

n/a	Involved in the study	
[ ]	[x]	ChIP-seq
[ ]	[x]	Flow cytometry
[x]	[ ]	MRI-based neuroimaging

Antibodies

Antibodies used	Flow Cytometry:<br>Viability eflour506 eBioscience 65-0866-18<br>mouse CD45.1 APC-Cy7 Biolegend 103116 30-F11<br>CD45 AF700 eBioscience 56-9459-42 2D1<br>CD2 PerCP-Cy5.5 Biolegend 300216 RPA-2.10<br>CD3 PerCP-Cy5.5 Biolegend 317336 okt3<br>CD3 BV711 Biolegend 317327 okt3<br>CD14 PerCP-Cy5.5 Biolegend 301824 M5E2<br>CD16 PerCP-Cy5.5 Biolegend 302028 3G8<br>CD56 PerCP-Cy5.5 Biolegend 318322 HCD56<br>CD56 BV605 Biolegend 318334 HCD56<br>CD235a PerCP-Cy5.5 Biolegend 318322 HIR2<br>CD34 BV421 Biolegend 343610 561<br>CD34 PE-Cy7 eBioscience 25-0349-42 4H11aka8G12<br>CD38 BV605 Biolegend 303532 HIT2<br>CD19 APC Biolegend 302212 HIB19<br>CD10 PE-Cy7 eBioscience 25-0106-42 ebioCB-CALLA<br>CD20 eflou450 eBioscience 48-0209-42 2H7<br>IgM FITC Biolegend 314506 MHM-88<br>IgD FITC biolegend 348205 IA6-2<br>CD133 PE Miltenyi 130-080-801 AC133<br>CD20 APC BD 641414<br>CD10 FITC eBioscience 11-0118-42 ICRF44<br>CD19 APC BD SJ25C1<br>CD24 BV510 Pharmagen 563035<br>CD45 V450 BD 642275<br>CD34 PerCp BD 345803<br>CD38 FITC BD 340909<br>NG2 PE Beckman Coulter B92429<br><br>ChIP-seq:<br>MLL-N Bethyl A300-086A<br>AF4-C Abcam ab31812<br>H3K79me2 Millipore 04-835 NL59
Validation	Flow cytometry antibodies validated by titration in-house using primary human fetal mononuclear cells (MNC) or NSG mouse bone marrow cells.<br>ChIP-seq antibodies were validated for ChIP in-house by ChIP-qPCR using the SEM cell line.

Animals and other organisms

Policy information about studies involving animals; ARRIVE guidelines recommended for reporting animal research

Laboratory animals	NOD.Cg-PrkdcscidIl2rgtm1Wjl/SzJ (NSG) mice, Jackson Labs (005557). Transplanted animals were all females aged 8-12 weeks at time of transplantation.
Wild animals	None
Field-collected samples	None
Ethics oversight	All experiments were performed under a project license approved by the UK Home Office under the Animal (Scientific Procedures) Act 1986 after approval by the Oxford Clinical Medicine Animal Welfare and Ethical Review Body; and in accordance with the principles of 3Rs (replacement, reduction and refinement) in animal research.

Note that full information on the approval of the study protocol must also be provided in the manuscript.

Human research participants

Policy information about studies involving human research participants

Population characteristics	Primary fetal liver hematopoietic cells from 13-15 post-conception week human fetuses were used in this study; 4 male and 2 female.<br>Acute leukemia patient samples were obtained from Blood Cancer UK Childhood Leukaemia Cell Bank, UK. All samples were anonymised at source, provided a unique study number and linked. The only population characteristic available to us was age. 3 patient samples from the UK CellBank were analyzed (ages: 6-18 years). Patient data was similarly anonymised at

	source for Great Ormond Street Hospital diagnostic samples. 11 patients' diagnostic data was included (age range: 6 months-11 years)
Recruitment	Donated fetal tissue was provided for purposes of this research by the Human Developmental Biology Resource (HDBR, www.hdbr.org), regulated by the UK Human Tissue Authority (HTA, www.hta.gov.uk) and covered under ethics (REC: 18/NE/0290 and 18/LO/0822). Informed consent was obtained from all participants, who donated human fetal tissue for research without receiving any monetary compensation. Donors were chosen based on gestational age.<br>Acute leukemia patient samples were obtained from Blood Cancer UK Childhood Leukaemia Cell Bank, UK after appropriate review of our research project to ensure that it was covered under their ethics approval (REC: 16/SW/0219). Informed consent was obtained from all participants or those with parental responsibility, and participants did not receive any monetary compensation.
Ethics oversight	UK Human Tissue Authority (HTA, www.hta.gov.uk). Ethical approval: NHS HRA: REC: 16/SW/0219, 18/NE/0290 and 18/LO/0822

Note that full information on the approval of the study protocol must also be provided in the manuscript.

ChIP-seq

Data deposition

- [x] Confirm that both raw and final processed data have been deposited in a public database such as GEO.
- [x] Confirm that you have deposited or provided access to graph files (e.g. BED files) for the called peaks.

Data access links<br>*May remain private before publication.*	To review GEO accession GSE162041:<br>Go to https://www.ncbi.nlm.nih.gov/geo/query/acc.cgi?acc=GSE162041<br>Enter token wxchwooejrwnbaf into the box
Files in database submission	crisprMLL-AF4_rep1_R1.fastq.gz<br>crisprMLL-AF4_rep2_R1.fastq.gz<br>crisprMLL-AF4_rep3_R1.fastq.gz<br>control_rep1_R1.fastq.gz<br>control_rep2_R1.fastq.gz<br>control_rep3_R1.fastq.gz<br>FL_HSC_rep1_R1.fastq.gz<br>FL_HSC_rep2_R1.fastq.gz<br>FL_HSC_rep3_R1.fastq.gz<br>FL_MPP_rep1_R1.fastq.gz<br>FL_MPP_rep2_R1.fastq.gz<br>FL_MPP_rep3_R1.fastq.gz<br>FL_LMPP_rep1_R1.fastq.gz<br>FL_LMPP_rep2_R1.fastq.gz<br>FL_LMPP_rep3_R1.fastq.gz<br>FL_CBP_rep1_R1.fastq.gz<br>FL_CBP_rep2_R1.fastq.gz<br>crisprMLL-AF4_rep1_R2.fastq.gz<br>crisprMLL-AF4_rep2_R2.fastq.gz<br>crisprMLL-AF4_rep3_R2.fastq.gz<br>control_rep1_R2.fastq.gz<br>control_rep2_R2.fastq.gz<br>control_rep3_R2.fastq.gz<br>FL_HSC_rep1_R2.fastq.gz<br>FL_HSC_rep2_R2.fastq.gz<br>FL_HSC_rep3_R2.fastq.gz<br>FL_MPP_rep1_R2.fastq.gz<br>FL_MPP_rep2_R2.fastq.gz<br>FL_MPP_rep3_R2.fastq.gz<br>FL_LMPP_rep1_R2.fastq.gz<br>FL_LMPP_rep2_R2.fastq.gz<br>FL_LMPP_rep3_R2.fastq.gz<br>FL_CBP_rep1_R2.fastq.gz<br>FL_CBP_rep2_R2.fastq.gz<br>crisprMLL-AF4_featureCounts.txt<br>FL_featureCounts.txt<br>crisprMLL-AF4_MLL-N_R1.fastq.gz<br>crisprMLL-AF4_AF4-C_R1.fastq.gz<br>crisprMLL-AF4_input_R1.fastq.gz<br>crisprMLL-AF4_H3K79me2_R1.fastq.gz<br>crisprMLL-AF4_H3K79me2_input_R1.fastq.gz<br>matched_FL_control_H3K79me2_R1.fastq.gz

matched_FL_control_H3K79me2_input_R1.fastq.gz
crisprMLL-AF4_MLL-N_R2.fastq.gz
crisprMLL-AF4_AF4-C_R2.fastq.gz
crisprMLL-AF4_input_R2.fastq.gz
crisprMLL-AF4_H3K79me2_R2.fastq.gz
crisprMLL-AF4_H3K79me2_input_R2.fastq.gz
matched_FL_control_H3K79me2_R2.fastq.gz
matched_FL_control_H3K79me2_input_R2.fastq.gz
crisprMLL-AF4_MLL-N.ucsc.bigWig
crisprMLL-AF4_AF4-C.ucsc.bigWig
crisprMLL-AF4_H3K79me2.ucsc.bigWig
matched_FL_control_H3K79me2.ucsc.bigWig
crisprMLL-AF4_MLL-AF4_peaks.bed
SEM_cell_MLL-AF4_peaks.bed
MLL-AF4_chALL_patient_MLL-AF4_peaks.bed

Genome browser session (e.g. UCSC): https://genome.ucsc.edu/s/siobhanrice01/Rice_etal_FL_MLLAF4

Methodology

Replicates: One replicate for ChIP-seq datasets

Sequencing depth:
CRISPRMLL-AF4+ MLL-N: total reads: 48201716, uniquely mapped reads: 25545996, read length: 40 bp, Paired end
CRISPRMLL-AF4+ AF4-C: total reads: 48685298, uniquely mapped reads: 24622992, read length: 40 bp, Paired end
CRISPRMLL-AF4+ H3K79me2: total reads: 8268490, uniquely mapped reads: 3190428, read length: 40 bp, Paired end
matched FL control H3K79me2: total reads: 7193046, uniquely mapped reads: 642686, read length: 40 bp, Paired end

Antibodies:
MLL-N Bethyl A300-086A
AF4-C Abcam ab31812
H3K79me2 Millipore 04-835

Peak calling parameters: Peaks were called using the Homer tool findPeaks, with the input track provided for background correction, using the -style histone or -style factor options to call peaks in histone modification or MLL-N/AF4-C datasets, respectively.

Data quality: Called peaks were analyzed as described on homer.ucsd.edu and compared to input track, with a threshold of FDR < 0.001 applied.

Software: Quality control of FASTQ reads, genome alignment, PCR duplicate filtering, blacklisted region filtering and UCSC data hub generation was performed using an in-house pipeline (Telenius et al., 2018. Reference 53 in manuscript).

Flow Cytometry

Plots

Confirm that:

- [x] The axis labels state the marker and fluorochrome used (e.g. CD4-FITC).
- [x] The axis scales are clearly visible. Include numbers along axes only for bottom left plot of group (a 'group' is an analysis of identical markers).
- [x] All plots are contour plots with outliers or pseudocolor plots.
- [x] A numerical value for number of cells or percentage (with statistics) is provided.

Methodology

Sample preparation: Samples analyzed by flow cytometry include CRISPRMLL-AF4-edited or mock-edited control primary human FL hematopoietic cells after either (i) co-culture on MS-5 stroma or (ii) transplantation in NSG mice (data shown for PB and BM). For PB flow cytometry from NSG mice, cells first underwent RBC lysis using ammonium chloride. Cells were stained with fluorophore-conjugated monoclonal antibodies in PBS with 2% FBS and 1mM EDTA for 30 minutes.

Instrument: Cells were analyzed on a Fortessa X50 instrument

Software: Data were collected using BDFACSDiva software (version 8.0.2). Analysis was performed using FlowJo software (version 10.7.1).

Cell population abundance: Post-sort purity checks were not carried out for sorted human CD45+CD19+ cells used for RT-qPCR and RNA-seq.

Gating strategy: A broad morphology gate was first set using FSC-A/SSC-A. Single cells were gated using FSC-A/FSC-H, followed by SSC-A/SSC-H. Fluorescence gates were set using unstained and fluorescence minus one controls.

- [x] Tick this box to confirm that a figure exemplifying the gating strategy is provided in the Supplementary Information.
